# Evaluating the risk and risk factors of dysautonomia as a post-acute sequelae of COVID-19: a secondary analysis of a matched case–control dataset

**DOI:** 10.3389/fneur.2025.1653175

**Published:** 2025-10-14

**Authors:** Benjamin C. Pierson, Megan Clare Craig-Kuhn, Laveta Stewart, Erica Sercy, Caryn A. Stern, Brock Graham, Amber Michel, Edward Parmelee, Tracey Perez Koehlmoos, David Saunders, James D. Mancuso, Simon Pollett, Timothy Burgess, David R. Tribble

**Affiliations:** ^1^Department of Preventive Medicine and Biostatistics, Uniformed Services University of the Health Sciences, Bethesda, MD, United States; ^2^Infectious Disease Clinical Research Program, Department of Preventive Medicine and Biostatistics, Uniformed Services University of the Health Sciences, Bethesda, MD, United States; ^3^Henry M. Jackson Foundation for the Advancement of Military Medicine, Inc., Bethesda, MD, United States; ^4^Joint Trauma System, Joint Base San Antonio, San Antonio, TX, United States; ^5^Department of Medicine, Uniformed Services University of the Health Sciences, Uniformed Services University, Bethesda, MD, United States

**Keywords:** COVID-19, long Covid, post-acute sequela of COVID-19, dysautonomia, case-control study, US military, autonomic disorders

## Abstract

**Background:**

A significant proportion of patients presenting with post-acute sequelae of COVID-19 (PASC) have been found to meet diagnostic criteria for certain disorders of the autonomic nervous system. Substantial gaps remain in our understanding of these conditions. Our objective is to evaluate demographic and medical factors associated with PASC dysautonomia in active duty US Service members (ADSM). Additionally we assessed for risk factors in those diagnosed with COVID-19 for PASC dysautonomia, and differences in those with PASC dysautonomia and non-PASC dysautonomia.

**Methods:**

A matched case control dataset (*n* = 1,367,961) of ADSM diagnosed with COVID-19 matched with ADSM with no evidence of COVID-19 was utilized to assess associations of demographic and clinical factors with PASC dysautonomia. Logistic regression modeling was used to assess differences among those diagnosed with COVID-19. Conditional logistic regression modeling using propensity score weighting was used for comparisons between those with PASC dysautonomia and non-PASC dysautonomia.

**Results:**

We identified 619,983 COVID-19 cases (158 PASC dysautonomia) and 747,978 controls (219 non-PASC dysautonomia). Among COVID-19 cases, factors positively associated with PASC dysautonomia were white, non-Hispanic race/ethnicity, female sex, younger age, northeast region, more severe COVID-19 infection, and comorbid depression or anxiety. Among those with dysautonomia, those with PASC dysautonomia were more likely to be of female sex, younger, in the northeast region, and less likely to have comorbid anxiety.

**Conclusion:**

PASC dysautonomia is rare in ADSM but associated with increased care utility and often prolonged diagnostic pathways. Important demographic and COVID-19 specific risk factors are associated with the development of PASC dysautonomia. PASC dysautonomia has significant differences in risk factors as compared to non-PASC dysautonomia, warranting further examination. These findings may support clinician awareness and prognostication and prompt further research on the pathophysiology and management of these conditions.

## Introduction

The COVID-19 pandemic has resulted in significant harms to the health of the world’s population and economy (1–3). To date the WHO has identified over 750 million diagnoses of COVID-19, resulting in over 7 million deaths (4). While recent strains of the SARS-COV-2 virus which causes COVID-19 has been associated with less virulent infections, there is still substantial morbidity and mortality from this now endemic pathogen (5, 6).

Early in the COVID-19 pandemic, reports began to identify individuals with persistent symptoms and/or symptoms developing after resolution of acute disease related to their COVID-19 infection (7, 8). This condition has been referred to as long COVID, long-haul COVID, post COVID condition, and post-acute sequelae of COVID (PASC, which will be used henceforth) (9). Symptoms associated with this condition appear widely variable, and encompass sustained symptoms similar to those seen in the acute phase of infection including cough, fatigue, alterations in sensation of taste and smell, as well as other atypical symptoms that typically onset after the acute phase of COVID-19 has ended including cognitive and psychological problems (‘brain fog’), palpitations and other cardiac symptoms, and neurologic symptoms (10–13). This heterogeneity in symptoms raises questions regarding differences in underlying disease pathophysiology between differing phenotypes of PASC (14, 15). Some risk factors that have been identified for PASC include female sex, Hispanic ethnicity, older age, underlying health conditions, including depression and anxiety, more severe acute COVID-19 infection, and being unvaccinated for COVID-19 (15, 16).

In some studies of PASC patients, a significant proportion meet diagnostic criteria for certain dysautonomia conditions (17, 18). Dysautonomia, or conditions that affect the function of the autonomic nervous system, which controls involuntary systems including heart rate and blood flow, are recently becoming increasingly identified following the onset of the COVID-19 pandemic (19, 20). While the underlying pathophysiology of COVID-19 induced dysautonomia (“PASC dysautonomia) remains uncertain, several hypotheses have been proposed. These include direct viral effects on the autonomic nervous system potentiating disordered regulation of neurotransmitters including serotonin, dysregulated inflammatory responses affecting the autonomic nervous system, and viral induced endothelial dysfunction impairing vascular function (21–25). The interplay between serotonin and other neurotransmitters with PASC, dysautonomia, and PASC dysautonomia are of particular interest as these conditions are frequently comorbid with anxiety and depression (26–28). There are several major gaps in our understanding of PASC dysautonomia (29). First, the prevalence of PASC dysautonomia is unclear, including in the Military Health System (MHS). Second, it is unclear whether PASC dysautonomia has distinct risk factors and pathophysiological mechanisms than dysautonomia not associated with COVID-19 (“non-PASC-dysautonomia”), and if differing approaches to clinical prediction and management are warranted for these individuals (29–31).

The purpose of this large scale (*n* = 1,367,961) EMR study was to evaluate dysautonomia in a study of individuals diagnosed with COVID-19 matched with individuals with no evidence of COVID-19 in the MHS. Objectives of the study included identification of demographic, military Service, and health related factors among individuals diagnosed with COVID-19 that increased the likelihood of developing dysautonomia after COVID-19 diagnosis. A further objective was to compare risk factors between PASC dysautonomia and non-PASC dysautonomia. Finally, we estimated the impact of PASC-dysautonomia, compared to those with a history of COVID-19 and no dysautonomia, through an analysis of associated medical care utility.

## Methods

### Study population

The population under consideration in this study was derived from the COVID-19 Military Registry Analysis Project (M-RAP), which identified diagnoses of COVID-19 in the MHS through data pulls from the DoD Military Health System Data Repository (MDR) from January 1^st^, 2020, through June 30^th^, 2022. Diagnoses of COVID-19 were determined via either a confirmatory laboratory diagnosis or ICD coding suggestive of COVID-19 (included in Supplementary Table S1) (32). The subset of the data examined for this analysis consisted of all active duty Service members diagnosed with COVID-19 in this period; for each individual with a COVID-19 diagnosis identified up to three individuals without a COVID-19 diagnosis were selected matched on region of residence, age (+/− 2 years), and gender. Medical claims data, pharmacy, and laboratory data for everyone identified were accessed from January 2017 through June 2023. All individuals included in the dataset were active duty MHS beneficiaries when diagnosed (or during the same month as the matched case was diagnosed for controls) and have no missing data for the variables of interest for this analysis.

### Dysautonomia status

The presence of dysautonomia was determined based upon identification of medical encounters using an ICD code suggestive of a dysautonomia condition (a subset of G90 ICD codes, included in Supplementary Table S2). Probable diagnosis of dysautonomia was used for individuals with three encounters containing such codes, while a status of possible diagnosis of dysautonomia was used for those with at least one such encounter. Given the complex and likely disparate diagnostic pathways to a dysautonomia diagnosis in the MHS, no upper bound on the time from COVID-19 diagnosis to dysautonomia diagnosis was used to accommodate for potential late presentation to care and delays in diagnosis. For the purposes of evaluating dysautonomia in the setting of PASC in the subset of individuals diagnosed with COVID-19, the first encounter in which one of these codes had to be after the initial date of COVID-19 diagnosis for the participant to be considered to have PASC dysautonomia, whereas for controls without COVID-19 there was no limitation on the timing of dysautonomia.

### Covariates

For all individuals included in the analysis demographic and military service variables were considered including sex, age, race/ethnicity, rank, military Service, geographical region (assessed by grouped US Department of Health and Human Services regions, and international classifiers). Additionally, the presence of potential associated illnesses was considered including depression, anxiety, diabetes, heart disease, and autoimmune disease. In addition to these factors, for those diagnosed with COVID-19 additional factors were considered related to their COVID infection including severity of infection, vaccination status, variant era of infection, and medication use within 3 months of infection that may be used for COVID-19 treatment, or be suggestive of dysautonomia including alpha agonists, beta blockers, corticosteroids, selective serotonin reuptake inhibiters, or stimulants. Severity of infection was categorized into groups with no additional medical visits within 14 days of COVID-19 diagnosis, those with at least 2 additional medical visits within 14 days of COVID-19 diagnosis but were not hospitalized, and those who were hospitalized within 30 days of COVID-19 diagnosis. The medication types were identified using the American Society of Health-System Pharmacists therapeutic classification schema (33).

### Statistical analyses

**Figure 1:**
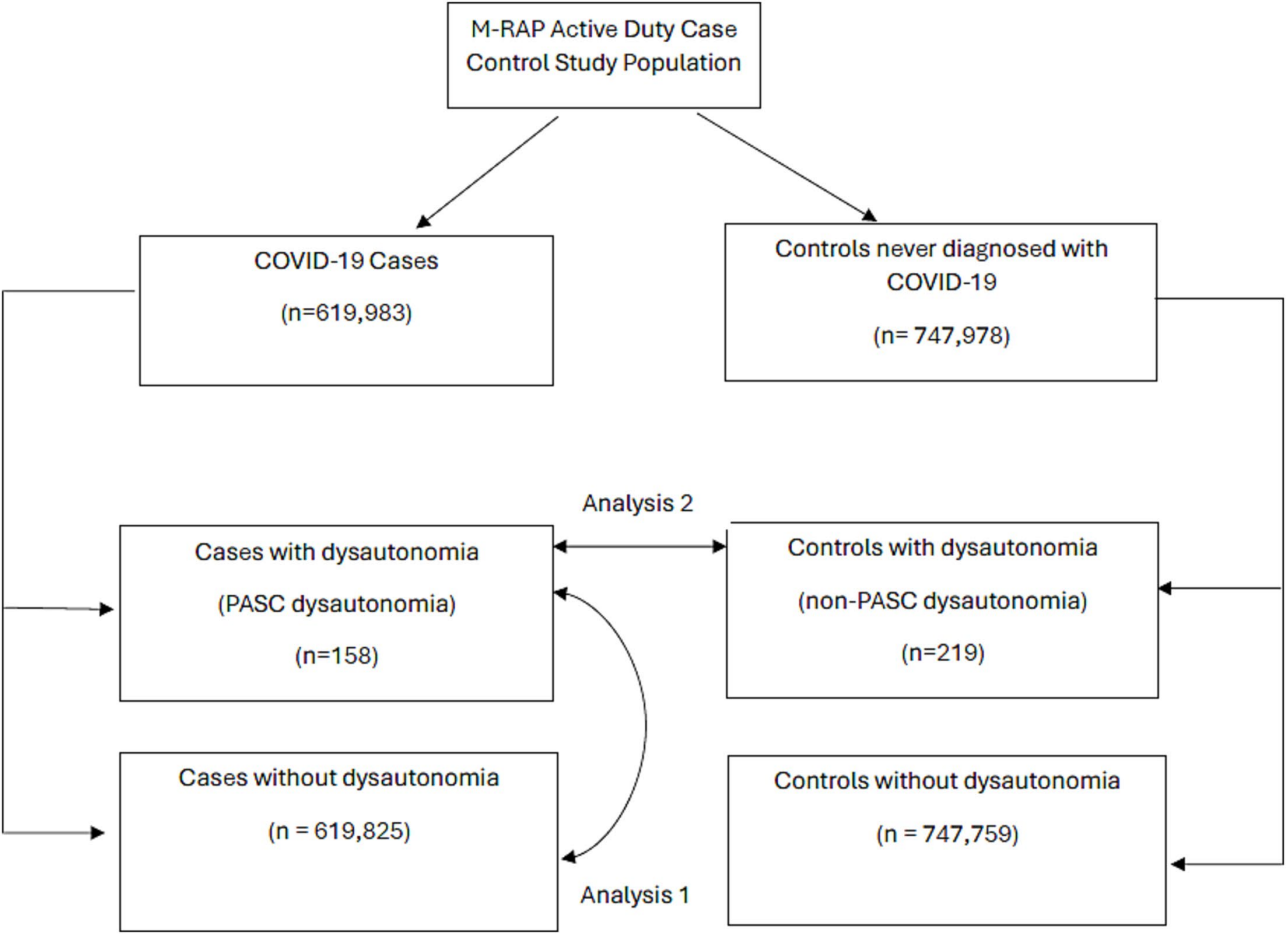
Flow diagram of military registry analysis project participants by COVID-19 case/control status, and dysautonomia status. Arrows are used to highlight the groups between which analysis 1 and analysis 2 were performed.

Two analyses were conducted on this sample; a flowchart showing the participant groups and analyses performed is presented (Figure 1). Directed Acyclic Graphs (DAGS, Supplementary Figures S1, S2) were constructed to visualize rationale in developing models to assess dysautonomia risk factors among these groups. These DAGs were used to identify possible covariates which may be included in multivariate regression models, with further statistical criteria used for covariate selection. The first analysis compared those with a COVID-19 infection who were diagnosed with dysautonomia following their COVID-19 infection (PASC dysautonomia) to those diagnosed with COVID-19 without a dysautonomia diagnosis to ascertain risk factors for the development of PASC dysautonomia in terms of patient demographics and COVID-19 infection related variables. Bivariate analyses using logistic regression was performed to identify initial associations between each variable and dysautonomia status among those diagnosed with COVID-19. Variables that had adequate numbers in each group and resulted in a statistically significant effect with at least a 20% change in the odds ratio of dysautonomia in the bivariate analyses were used to develop a full model of dysautonomia associations. The included variables were first analyzed through a correlation matrix to assess collinearity, with prespecified limit of correlation coefficients >0.7 needing to be interrogated and corrected if identified. The final variables were then used to construct a full logistic regression model. Given the concern for low numbers of dysautonomia diagnoses in the military, an additional sensitivity analysis using Firth’s penalized likelihood method was performed on this model.

The next analysis evaluated those with PASC dysautonomia to those with non-PASC dysautonomia to assess if there were significant differences in demographic risk groups for the development of these COVID-19 associated conditions as compared to traditional diagnoses. As the data came from a matched case control dataset of individuals diagnosed with COVID-19 and controls with no evidence of COVID-19 matched on age, sex, and region, this analysis utilized a propensity score weighting using inverse probability weights using all variables under consideration in developing propensity scores for each individual. The analysis was performed using logistical regression conditioned on the matched variables of age, sex, and region, and weighted using the inverse probability weights. This methodology was used in evaluating these groups to account for the matched design while also adjusting for potential confounding covariates which were not matched (34, 35). The validity of the propensity score weighting was evaluated using a covariate balance assessment of standard mean differences of covariates between groups. After applying the weighting, bivariate conditional logistic regression was performed assessing associations of demographic and comorbid medical conditions with PASC dysautonomia. Variables that had adequate numbers in each group and resulted in a statistically significant effect that resulted in at least a 20% change in the odds ratio of dysautonomia in the bivariate analyses were used to develop a full model of dysautonomia associations. The included variables were first analyzed through a correlation matrix to assess collinearity, with prespecified limit of correlation coefficients >0.7 needing to be interrogated and corrected if identified. The final variables were then used to construct a full conditional logistic regression model.

For all analyses, the primary assessment was made on participants deemed to have probable dysautonomia, and sensitivity analyses were performed using the definition of possible dysautonomia. Referent groups in analyses were generally selected as the group with the largest population, or at the lowest level of an ordinal variable. The goodness of fit of all regression models was assessed using the Hosmer-Lemeshow method. All analyses were conducted using SAS version 9.4 (SAS Institute Inc). This research received an exempt determination by the Uniformed Services University of the Health Sciences Human Research Protection Program.

## Results

### Demographics

**Figure 2:**
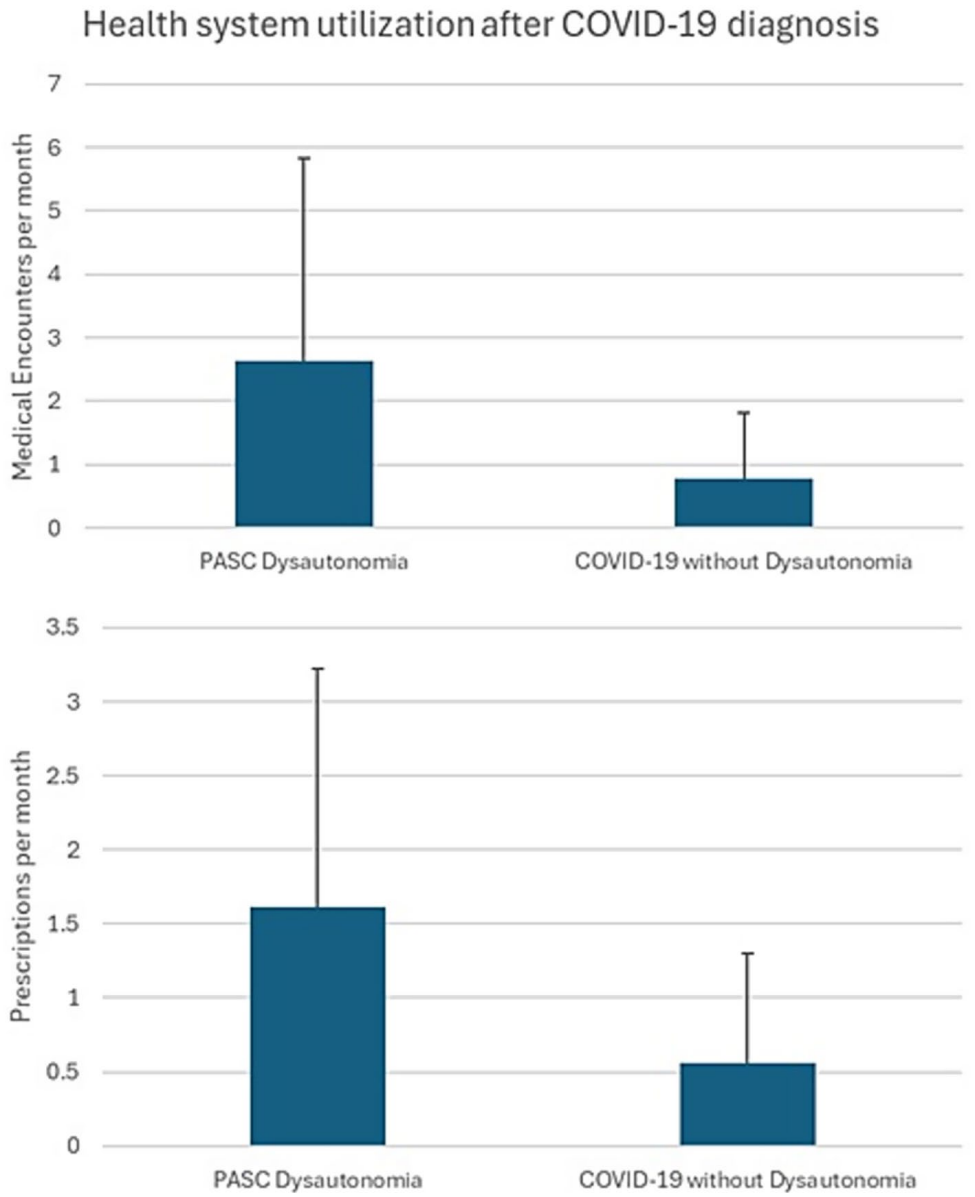
Military health system (MHS) utilization of individuals with PASC dysautonomia and those with COVID-19 infection never diagnosed with dysautonomia.

A total of 1,367,961 active duty Service members were included in the analysis, comprising 619,983 individuals diagnosed with COVID-19 and 747,978 individuals with no documented COVID-19 infection. A breakdown of the demographics, Service-related variables, and medical comorbidities of these individuals is presented (Table 1). A total of 158 individuals diagnosed with COVID-19 were identified as probable PASC dysautonomia (551 possible PASC dysautonomia), while 219 individuals without documented COVID-19 infection were identified as probable non-PASC dysautonomia (692 possible non-PASC dysautonomia). Breakdowns of the demographics, Service-related variables, and medical comorbidities of those with PASC dysautonomia as compared to the remainder of those diagnosed with COVID-19 and non-PASC dysautonomia as compared to the remainder of those without a COVID-19 diagnosis are presented (Tables 2, 3, respectively). Breakdowns of the variables relating to COVID-19 infection, including severity, variant era of infection and vaccination status, and medications prescribed within 90 days before infection are also presented (Table 2). The median time after COVID-19 infection for the first encounter in which a dysautonomia code was used was 186 days (IQR: 59 to 319 days). The MHS utilization of individuals with PASC dysautonomia and those with COVID-19 infection never diagnosed with dysautonomia is presented in Figure 2. Individuals with PASC dysautonomia had increased utilization of medical services after their COVID-19 diagnosis, having a mean of 2.63 (SD 3.20) medical encounters per month after their COVID-19 infection, as compared to 0.79 (SD 1.03) encounters per month after COVID-19 infection for those not diagnosed with dysautonomia. They also tended to be prescribed more medications, having prescriptions written for them at a rate of 1.61 (SD 1.61) prescriptions per month, as compared to 0.56 (SD 0.74) prescriptions per month after COVID-19 infection for those not diagnosed with dysautonomia.

**Table 1:** Breakdown of relevant demographic and medical variables in M-RAP study population between those with a documented COVID-19 infection and those without documented COVID-19 infection.

		M-RAP case–control population (*n* = 1,367,961)			
		COVID-19 case (*n* = 619,983)		No known COVID-19 (*n* = 747,978)	
		*n*	%	*n*	%
Sex	Male	487,556	78.64%	621,696	83.12%
	Female	132,427	21.36%	126,282	16.88%
Service	Air Force	172,053	27.75%	184,197	24.63%
	Army	252,801	40.78%	268,171	35.85%
	Marine Corps	65,637	10.59%	76,273	10.20%
	Navy	118,298	19.08%	198,090	26.48%
	Coast Guard	11,194	1.81%	21,247	2.84%
Rank	Junior Enlisted (E1-E4)	260,182	41.97%	197,543	26.41%
	Senior Enlisted (E5+)	258,930	41.76%	374,499	50.07%
	Junior Officer (O1-O3)	53,595	8.64%	80,237	10.73%
	Senior Officer (O4+)	38,342	6.18%	79,233	10.59%
	Warrant or Other	8,934	1.44%	16,466	2.20%
Age	<25	226,573	36.55%	187,629	25.08%
	25–34	241,961	39.03%	295,574	39.52%
	35–44	121,865	19.66%	202,099	27.02%
	> = 45	29,584	4.77%	62,676	8.38%
Race/Ethnicity	American Indian	7,401	1.19%	9,848	1.32%
	AAPI*	37,444	6.04%	47,448	6.34%
	Black, not hispanic	105,713	17.05%	111,516	14.91%
	Hispanic	115,064	18.56%	110,341	14.75%
	White, not hispanic	329,039	53.07%	427,694	57.18%
	Unknown/Other	25,322	4.08%	41,131	5.50%
Region**	Northeast	219,131	35.34%	275,835	36.88%
	South	52,724	8.50%	58,956	7.88%
	Midwest	156,281	25.21%	161,706	21.62%
	West	115,107	18.57%	159,182	21.28%
	Asia	29,778	4.80%	34,758	4.65%
	Europe	27,510	4.44%	32,862	4.39%
	Other	19,452	3.14%	24,679	3.30%
Comorbid conditions	Diabetes	2,958	0.48%	4,674	0.62%
	Depression	34,670	5.59%	38,330	5.12%
	Anxiety	62,980	10.16%	67,252	8.99%
	Autoimmune disease	2,368	0.38%	3,071	0.41%
	Heart disease	1978	0.32%	2,597	0.35%

*Asian American or Pacific Islander **US regions by HHS region(Northeast 1,2,3; South 4,6; Midwest 5,7,8; West 9,10), Other primarily includes Africa and Central/South America.

**Table 2:** Breakdown of relevant factors in COVID-19 cases between those with PASC dysautonomia and those without dysautonomia diagnosis.

	COVID Cases (*n* = 619,983)	PASC dysautonomia (*n* = 158)		No dysautonomia (*n* = 619,825)	
		*n*	%	*n*	%
Sex	Male	91	57.59%	487,465	78.65%
	Female	67	42.41%	132,360	21.35%
Service	Air Force	34	21.52%	172,019	27.75%
	Army	64	40.51%	252,737	40.78%
	Marine Corps	28	17.72%	65,609	10.59%
	Navy	30	18.99%	118,268	19.08%
	Coast Guard/Other	2	1.27%	11,192	1.81%
Rank	Junior Enlisted (E1-E4)	49	31.01%	260,133	41.97%
	Senior Enlisted (E5+)	81	51.27%	258,849	41.76%
	Junior Officer (O1-O3)	16	10.13%	53,579	8.64%
	Senior Officer (O4+)	10	6.33%	38,332	6.18%
	Warrant or other	2	1.27%	8,932	1.44%
Age	<25	45	28.48%	226,528	36.55%
	25–34	55	34.81%	241,906	39.03%
	35–44	52	32.91%	121,813	19.65%
	> = 45	6	3.80%	29,578	4.77%
Race/Ethnicity	American Indian	1	0.63%	7,400	1.19%
	AAPI*	5	3.16%	37,439	6.04%
	Black, not hispanic	15	9.49%	105,698	17.05%
	Hispanic	23	14.56%	115,041	18.56%
	White, not Hispanic	106	67.09%	328,933	53.07%
	Unknown/Other	8	5.06%	25,314	4.08%
Region**	Northeast	84	53.16%	219,047	35.34%
	South	12	7.59%	52,712	8.50%
	Midwest	16	10.13%	156,265	25.21%
	West	27	17.09%	115,080	18.57%
	Asia	7	4.43%	29,771	4.80%
	Europe	9	5.70%	27,501	4.44%
	Other	3	1.90%	19,449	3.14%
Comorbid conditions	Diabetes	0	0.00%	2,958	0.48%
	Depression	46	29.11%	34,624	5.59%
	Anxiety	64	40.51%	62,916	10.15%
	Autoimmune disease	2	1.27%	2,366	0.38%
	Heart disease	2	1.27%	1976	0.32%
Vaccination Status	Un /Partially vaccinated	17	10.76%	48,195	7.78%
	Full primary series	93	58.86%	388,193	62.63%
	At least one booster	48	30.38%	183,437	29.59%
Variant Era	Ancestral	69	43.67%	199,073	32.12%
	Delta	18	11.39%	93,892	15.15%
	Omicron	71	44.94%	326,860	52.73%
Severity	No follow-up	42	26.58%	270,733	43.68%
	> = 2 Outpatient Appts	109	68.99%	341,725	55.13%
	Hospitalized	7	4.43%	7,367	1.19%
Medications	alpha agonist	2	1.27%	887	0.14%
	beta blocker	18	11.39%	7,411	1.20%
	SSRI	42	26.58%	38,521	6.21%
	Steroid	4	2.53%	13,947	2.25%
	Stimulant	4	2.53%	1,595	0.26%

*Asian American or Pacific Islander **US regions by HHS (Northeast 1,2,3; South 4,6; Midwest 5,7,8; West 9,10), Other primarily Africa and Central/South America.

**Table 3:** Breakdown of studied demographic and medical variables in those with no history of COVID-19 infection, between those with non-PASC dysautonomia and those with no diagnosis of dysautonomia.

	Controls (*n* = 747,978)	Dysautonomia (*n* = 219)		No dysautonomia (*n* = 747,759)	
		*n*	%	*n*	%
Sex	Male	155	70.78%	621,541	83.12%
	Female	64	29.22%	126,218	16.88%
Service	Air Force	34	15.53%	184,163	24.63%
	Army	97	44.29%	268,074	35.85%
	Marine Corps	45	20.55%	76,228	10.19%
	Navy	42	19.18%	198,048	26.49%
	Coast Guard/Other	1	0.46%	21,246	2.84%
Rank	Junior Enlisted (E1-E4)	41	18.72%	197,502	26.41%
	Senior Enlisted (E5+)	119	54.34%	374,380	50.07%
	Junior Officer (O1-O3)	16	7.31%	80,221	10.73%
	Senior Officer (O4+)	31	14.16%	79,202	10.59%
	Warrant or Other	12	5.48%	16,454	2.20%
Age	<25	36	16.44%	187,593	25.09%
	25–34	57	26.03%	295,517	39.52%
	35–44	95	43.38%	202,004	27.01%
	> = 45	31	14.16%	62,645	8.38%
Race/Ethnicity	American Indian	3	1.37%	9,845	1.32%
	AAPI*	11	5.02%	47,437	6.34%
	Black, not Hispanic	17	7.76%	111,499	14.91%
	Hispanic	26	11.87%	110,315	14.75%
	White, not Hispanic	152	69.41%	427,542	57.18%
	Unknown/Other	10	4.57%	41,121	5.50%
Region**	Northeast	92	42.01%	275,743	36.88%
	South	18	8.22%	58,938	7.88%
	Midwest	40	18.26%	161,666	21.62%
	West	51	23.29%	159,131	21.28%
	Asia	8	3.65%	34,750	4.65%
	Europe	9	4.11%	32,853	4.39%
	Other	1	0.46%	24,678	3.30%
Comorbid conditions	Diabetes	4	1.83%	4,670	0.62%
	Depression	77	35.16%	38,253	5.12%
	Anxiety	111	50.68%	67,141	8.98%
	Autoimmune Disease	2	0.91%	3,069	0.41%
	Heart Disease	3	1.37%	2,594	0.35%

*Asian American or Pacific Islander **US regions by HHS (Northeast 1,2,3; South 4,6; Midwest 5,7,8; West 9,10), Other primarily includes Africa and Central/South America.

### Analysis 1: PASC dysautonomia among those with COVID-19 infection

The bivariate analyses evaluating factors among those with COVID-19 infection associated with probable PASC dysautonomia are presented (Table 4). The majority of variables evaluated were found to have significant associations with PASC dysautonomia with the exception of comorbid heart disease, diabetes, autoimmune-related disease, prescription of corticosteroids before COVID infection, and vaccination status. Strikingly, the severity of COVID-19 infection showed a strong association with risk of dysautonomia that increased with the level of severity; additionally in relation to variant era of infection, with earlier variant eras of COVID-19 (prior to Delta variant) more associated with PASC dysautonomia. Further findings related to demographic status including higher risk among those who were white, non-Hispanic, women, aged 35–44, or residing in the Northeast. Prevalence of PASC dysautonomia among Marines also appears to be higher than other Services, and no significant effect associated with rank appears to be present. The prescription of beta blockers, alpha agonists, selective serotonin reuptake inhibitors, or stimulants within 90 days before COVID-19 infection also appears to have a strong positive association with PASC dysautonomia, along with the comorbid conditions of depression and anxiety. The results of the sensitivity analyses conducted following the same approach using the definition of possible PASC dysautonomia are presented (Table 5). Differences noted in the bivariate analyses include additional increased association with PASC dysautonomia in the >45 age group, in Service members in the Army, in ranks above junior enlisted, and among those with a history of heart disease or autoimmune disease and prescribed stimulants or steroids prior to COVID infection. Additionally, receiving the full primary series of COVID-19 vaccination has a protective effect in this group, however those with a booster dose did not have increased protection. In the full model many of these significant findings were attenuated to the point of being non-statistically significant, including the increased odds of PASC dysautonomia in the >45 age group, and ranks other than senior enlisted, comorbid autoimmune disease, concomitant use of steroids, as well as the protective effect of vaccination noted in the bivariate analysis. The results of the sensitivity analysis using the Firth’s penalized likelihood approach are provided in the Supplementary Table S3, with no deviations of note from our primary analysis.

**Table 4:** Logistic regression of probable PASC dysautonomia vs. COVID-19 cases without dysautonomia.

		Bivariate analysis			Full model		
		OR	95% CI		OR	95% CI	
Sex	Male	ref			ref		
	Female	2.712	1.978	3.718	2.436	1.737	3.417
Age	<25	0.874	0.589	1.296	0.962	0.582	1.589
	25–34	ref			ref		
	35–44	1.878	1.285	2.743	1.672	1.11	2.519
	> = 45	0.892	0.384	2.072	0.775	0.317	1.896
Race/Ethnicity	American Indian	0.419	0.059	3.005	0.322	0.045	2.331
	AAPI*	0.414	0.169	1.016	0.393	0.159	0.969
	Black, not Hispanic	0.44	0.256	0.756	0.317	0.183	0.552
	Hispanic	0.62	0.395	0.974	0.567	0.359	0.896
	White, not Hispanic	ref			ref		
	Unknown/Other	0.981	0.478	2.012	0.952	0.457	1.982
Rank	Junior Enlisted (E1-E4)	ref			ref		
	Senior Enlisted (E5+)	1.661	1.165	2.369	1.226	0.743	2.021
	Junior Officer (O1-O3)	1.585	0.902	2.788	1.335	0.696	2.56
	Senior Officer (O4+)	1.385	0.702	2.734	0.85	0.367	1.969
	Warrant or other	1.189	0.289	4.889	0.663	0.15	2.94
Service	Air force	ref			ref		
	Army	1.281	0.845	1.942	1.39	0.905	2.135
	Marine Corps	2.159	1.309	3.561	2.433	1.426	4.151
	Navy	1.283	0.785	2.097	0.976	0.586	1.627
	Coast Guard/Other	0.904	0.217	3.764	0.87	0.206	3.675
Region**	Northeast	ref			ref		
	South	0.594	0.324	1.087	0.654	0.353	1.214
	Midwest	0.267	0.156	0.456	0.319	0.183	0.555
	West	0.612	0.397	0.944	0.922	0.573	1.483
	Asia	0.613	0.284	1.326	0.878	0.397	1.938
	Europe	0.853	0.429	1.697	1.061	0.526	2.141
	Other	0.402	0.127	1.273	0.745	0.232	2.395
Vaccination status	Un/Partial vaccination	ref					
	Full primary series	0.679	0.405	1.139			
	At least one booster dose	0.742	0.427	1.29			
Variant era	Ancestral	ref			ref		
	Delta	0.553	0.329	0.929	0.532	0.315	0.899
	Omicron	0.627	0.45	0.873	0.618	0.434	0.882
Severity	1 outpatient visit	ref			ref		
	> = 2 outpatient	2.056	1.44	2.935	1.463	0.976	2.192
	Hospitalization	6.126	2.751	13.639	2.981	1.294	6.865
Comorbid conditions	Diabetes	-	-	-			
	Depression	6.942	4.924	9.786	2.63	1.746	3.962
	Anxiety	6.027	4.386	8.281	2.489	1.696	3.653
	Heart disease	3.346	0.829	13.506			
	Autoimmune disease	4.009	0.993	16.182			
Medication use	Alpha agonist	8.949	2.216	36.148	8.639	2.069	36.079
	Beta blockers	10.629	6.504	17.371	3.583	2.062	6.224
	Steroid	1.128	0.418	3.045			
	SSRI	5.464	3.838	7.778	1.58	1.019	2.449
	Stimulant	10.068	3.727	27.199	3.609	1.309	9.949

Bivariate analysis and full model including all variables found to have a significant effect in bivariate analysis. *Asian American or Pacific Islander **US regions by HHS (Northeast 1,2,3; South 4,6; Midwest 5,7,8; West 9,10), Other primarily includes Africa and Central/South America.

**Table 5:** Logistic regression of possible PASC dysautonomia vs. COVID-19 cases without dysautonomia.

		Bivariate analysis			Full model		
		OR	95% CI		OR	95% CI	
Sex	Male	ref			ref		
	Female	2.563	2.162	3.037	2.411	2.01	2.892
Age	<25	0.789	0.629	0.989	1.046	0.785	1.394
	25–34	ref			ref		
	35–44	2.225	1.815	2.727	1.749	1.403	2.18
	> = 45	2.233	1.623	3.073	1.409	0.982	2.022
Race/Ethnicity	American Indian	0.593	0.245	1.432	0.515	0.212	1.253
	AAPI*	0.539	0.354	0.821	0.517	0.338	0.791
	Black, not Hispanic	0.415	0.309	0.557	0.298	0.221	0.404
	Hispanic	0.511	0.394	0.662	0.5	0.384	0.652
	White, not Hispanic	ref			ref		
	Unknown/Other	1.074	0.745	1.55	1.112	0.764	1.62
Rank	Junior Enlisted (E1-E4)	ref			ref		
	Senior Enlisted (E5+)	2.196	1.792	2.629	1.558	1.171	2.074
	Junior Officer (O1-O3)	1.714	1.233	2.382	1.315	0.901	1.917
	Senior Officer (O4+)	3.148	2.335	4.244	1.428	0.951	2.145
	Warrant or Other	1.499	0.701	3.206	0.723	0.324	1.614
Service	Air Force	ref			ref		
	Army	1.249	1.016	1.536	1.397	1.125	1.733
	Marine Corps	1.037	0.759	1.418	1.431	1.029	1.991
	Navy	0.994	0.766	1.29	0.872	0.663	1.146
	Coast Guard/Other	0.775	0.363	1.655	0.681	0.315	1.472
Region**	Northeast	ref			ref		
	South	0.593	0.428	0.823	0.609	0.436	0.85
	Midwest	0.439	0.347	0.557	0.493	0.384	0.632
	West	0.418	0.318	0.549	0.66	0.492	0.885
	Asia	0.538	0.345	0.838	0.734	0.464	1.16
	Europe	0.971	0.684	1.38	1.008	0.701	1.45
	Other	0.549	0.321	0.939	0.901	0.522	1.556
Vaccination status	Un/Partial vaccination	ref			ref		
	Full primary series	0.708	0.526	0.954	0.87	0.64	1.183
	At least one booster dose	1.077	0.793	1.463	1.055	0.763	1.46
Variant era	Ancestral	ref			ref		
	Delta	0.77	0.593	0.999	0.734	0.563	0.957
	Omicron	0.752	0.628	0.902	0.735	0.604	0.895
Severity	No outpatient	ref			ref		
	> = 2 outpatient	2.16	1.782	2.618	1.497	1.208	1.856
	Hospitalization	5.703	3.638	8.941	2.791	1.748	4.459
Comorbid conditions	Diabetes	1.913	0.792	4.616			
	Depression	4.563	3.719	5.598	1.639	1.295	2.075
	Anxiety	5.632	4.744	6.686	2.569	2.172	3.256
	Heart disease	6.834	4.014	11.636	2.948	1.688	5.147
	Autoimmune disease	4.62	2.296	9.297	1.837	0.9	3.753
Medication use	Alpha agonist	13.007	6.935	24.398	12.602	6.575	24.153
	Beta blockers	9.204	6.96	12.172	3.333	2.438	4.557
	Steroid	1.894	1.247	2.877	1.338	0.877	2.041
	SSRI	4.301	3.516	5.26	1.361	1.067	1.736
	Stimulant	7.935	4.361	14.439	3.167	1.718	5.837

Bivariate analysis and full model including all variables found to have a significant effect in bivariate analysis. *Asian American or Pacific Islander **US regions by HHS (Northeast 1,2,3; South 4,6; Midwest 5,7,8; West 9,10), Other primarily includes Africa and Central/South America.

### Analysis 2: PASC dysautonomia and non-PASC dysautonomia

The bivariate analyses evaluating factors associated with PASC dysautonomia as compared to non-PASC dysautonomia are presented (Table 6). Those 45 and older, of senior officer rank and residing in the Midwest had significantly lower odds. No differences in sex, race/ethnicity, or Service were noted. Strikingly, in individuals with dysautonomia those with anxiety had 60.4% of the odds of PASC dysautonomia as compared to non-PASC dysautonomia, suggesting that anxiety is significantly less prevalent in those with PASC dysautonomia, with 40.51% of those with PASC dysautonomia having comorbid anxiety as compared to 50.68% of those with non-PASC dysautonomia. In the full model, the effect of rank was attenuated to not significant levels, and the general trends seen in the bivariate analysis persisted throughout, with augmentation of the effect size seen in those with anxiety. The results of these analyses using the sensitivity analyses definition of possible dysautonomia are presented (Table 7). In these analyses, women had increased odds of PASC dysautonomia, those aged 35–44 and greater than 45 had decreased odds, with no significant effects by race/ethnicity, and those residing in Asia or other (Africa / South America) were noted to have higher odds of PASC dysautonomia. Regarding Service specific variables, those in the Army and of Warrant Officer or other rank had decreased odds of PASC dysautonomia. Those with depression or anxiety also had decreased odds. In the full model many of these effects were attenuated, with no significant findings by military Service or comorbid anxiety.

**Table 6:** Propensity score weighted conditional logistic regression of probable PASC dysautonomia vs. probable non-PASC dysautonomia.

		Bivariate analysis			Full model		
		OR	95% CI		OR	95% CI	
Sex	Male	ref					
	Female	1.458	0.957	2.222			
Age	<25	1.022	0.576	1.813	1.184	0.557	2.519
	25–34	ref			ref		
	35–44	0.648	0.397	1.058	0.62	0.353	1.09
	> = 45	0.262	0.108	0.634	0.258	0.093	0.714
Race/Ethnicity	American Indian	0.59	0.059	5.918			
	AAPI*	0.666	0.217	2.046			
	Black, not Hispanic	1.218	0.581	2.555			
	Hispanic	1.08	0.578	2.018			
	White, not Hispanic	ref					
	Unknown/Other	1.269	0.483	3.333			
Rank	Junior Enlisted (E1-E4)	ref			ref		
	Senior Enlisted (E5+)	0.813	0.497	1.33	1.259	0.599	2.646
	Junior Officer (O1-O3)	1.236	0.546	2.801	1.506	0.583	3.89
	Senior Officer (O4+)	0.438	0.199	0.962	0.952	0.319	2.846
	Warrant or Other	0.222	0.046	1.063	0.529	0.085	3.298
Service	Air Force	ref					
	Army	0.712	0.414	1.224			
	Marine Corps	0.694	0.358	1.345			
	Navy	0.882	0.465	1.672			
	Coast Guard/Other	3.115	0.267	36.389			
Region**	Northeast	ref			ref		
	South	0.653	0.296	1.438	0.573	0.238	1.378
	Midwest	0.388	0.199	0.758	0.35	0.174	0.704
	West	0.594	0.343	1.026	0.493	0.272	0.893
	Asia	1.075	0.531	2.178	0.941	0.38	2.33
	Europe	1.053	0.441	2.514	0.913	0.365	2.279
	Other	3.439	2.547	4.643	3.427	2.341	5.017
Comorbid conditions	Depression	0.684	0.439	1.066			
	Anxiety	0.604	0.398	0.915	0.556	0.355	0.873
	Diabetes	–	–	–			
	Heart disease	0.886	0.145	5.424			
	Autoimmune disease	1.385	0.188	10.189			

Bivariate analysis and full model including all variables found to have a significant effect and resulting in 20% change to OR in bivariate analysis. *Asian American or Pacific Islander **US regions by HHS (Northeast 1,2,3; South 4,6; Midwest 5,7,8; West 9,10), Other primarily includes Africa and Central/South America.

**Table 7:** Propensity score weighted conditional logistic regression of possible PASC dysautonomia vs. possible non-PASC dysautonomia.

		Bivariate analysis			Full model		
		OR	95% CI		OR	95% CI	
Sex	Male	ref			ref		
	Female	1.718	1.349	2.187	1.563	1.181	2.069
Age	<25	0.856	0.609	1.203	0.944	0.597	1.494
	25–34	ref			ref		
	35–44	0.558	0.42	0.741	0.608	0.441	0.839
	> = 45	0.436	0.296	0.644	0.482	0.303	0.766
Race/Ethnicity	American Indian	0.626	0.209	1.88			
	AAPI*	1.208	0.674	2.165			
	Black, not Hispanic	1.112	0.738	1.677			
	Hispanic	1.028	0.716	1.475			
	White, not Hispanic	ref					
	Unknown/Other	1.576	0.919	2.703			
Rank	Junior Enlisted (E1-E4)	ref					
	Senior Enlisted (E5+)	0.848	0.633	1.137	1.362	0.86	2.158
	Junior Officer (O1-O3)	1.157	0.714	1.875	1.337	0.748	2.39
	Senior Officer (O4+)	0.787	0.529	1.171	1.319	0.733	2.373
	Warrant or Other	0.2	0.086	0.463	0.362	0.142	0.918
Service	Air Force	ref			ref		
	Army	0.68	0.509	0.909	0.873	0.636	1.198
	Marine Corps	0.741	0.481	1.14	0.9	0.552	1.466
	Navy	0.974	0.675	1.405	0.954	0.63	1.443
	Coast Guard/Other	1.817	0.565	5.84	2.357	0.616	9.022
Region**	Northeast	ref			ref		
	South	1.263	0.796	2.002	1.054	0.64	1.737
	Midwest	0.939	0.684	1.289	0.8	0.559	1.145
	West	0.889	0.629	1.257	0.802	0.537	1.198
	Asia	2.093	1.048	4.178	2.009	0.948	4.258
	Europe	1.367	0.833	2.243	1.101	0.639	1.898
	Other	4.864	1.72	13.754	4.173	1.511	11.526
Comorbid conditions	Depression	0.676	0.519	0.881	0.719	0.536	0.965
	Anxiety	0.771	0.613	0.969	0.795	0.613	1.03
	Diabetes	0.575	0.194	1.702			
	Heart disease	1.119	0.539	2.323			
	Autoimmune disease	2.508	0.752	8.37			

Bivariate analysis and full model including all variables found to have a significant effect and resulting in 20% change to OR in bivariate analysis. *Asian American or Pacific Islander **US regions by HHS (Northeast 1,2,3; South 4,6; Midwest 5,7,8; West 9,10), Other primarily includes Africa and Central/South America.

## Discussion

Our analysis of 1,367,91 active duty Service members identified 158 individuals with probable PASC dysautonomia diagnosed with COVID-19, as well as 219 individuals with probable non-PASC dysautonomia in those without evidence of COVID-19 infection. Factors that appeared to place individuals infected with COVID-19 at higher risk of PASC dysautonomia included female sex, age 35–44, service in the Marine Corps, residing in the Northeast, severity of infection, infection with COVID-19 prior to the delta variant era of SARS-CoV-2, comorbid depression or anxiety, and prescription of beta blockers, stimulants, selective serotonin reuptake inhibitors, or alpha agonists prior the time of their COVID-19 infection. Individuals with PASC dysautonomia as compared to those with non-PASC dysautonomia also were more likely to be residing in the Northeast, and were less likely to be above age 45, and to have comorbid anxiety. These risk factors may assist clinicians predict those more likely to develop this complication.

While an uncommon complication, we noted that those with PASC dysautonomia had markedly higher medical utilization after their COVID-19 diagnosis as compared to those diagnosed with COVID-19 without a PASC dysautonomia diagnosis, with the lowest medical utilization seen in those without documented COVID-19 infection without dysautonomia. These findings support that PASC dysautonomia is a risk for increased medical utilization within the MHS, and additionally that COVID-19 diagnosis in general also has some impact on increasing medical utilization rates after diagnosis. Those diagnosed with PASC dysautonomia had a median time from COVID-19 infection to dysautonomia diagnosis of approximately 6 months, contrasting to a previous study of individuals with a specific form of dysautonomia (postural orthostatic tachycardia syndrome), who reported a median time from symptom onset to diagnosis of 24 months (36). The relatively faster diagnoses of the individuals in our sample could be due to the increased visibility and attention to PASC syndromes during the height of the COVID-19 pandemic. Time to diagnosis may be further reduced with augmented messaging to MHS providers to raise awareness of this significant COVID-19 complication.

The findings of increased likelihood of PASC dysautonomia with increasing severity of infection are consistent with several other studies on PASC (37–40). Our analysis also indicated that earlier variant eras of SARS-CoV-2 may have presented higher likelihood of PASC dysautonomia; this may be consistent with earlier variants of SARS-CoV-2 being associated with more severe illness, or potentially be due to earlier infections accommodating longer study follow-up time to identify PASC cases (41). While the effect of vaccination was only found to be significant in the bivariate sensitivity analysis (and potentially impacted by confounding), other studies have noted that vaccination status may confer some protection against PASC dysautonomia through attenuation of risk for severe acute COVID-19 disease (7, 15).

Demographic factors affecting the likelihood of PASC dysautonomia have some overlap with other studies, most consistently showing that those with female sex, and white, non-Hispanic race/ethnicity having higher prevalence of PASC (16, 42, 43). We noted an association of PASC dysautonomia with residence in the Northeast. Potential rationale for these differences may include the geographic dispersion of active duty military personnel, with those Service members residing in the Northeast being otherwise different on important risk factors from those in other regions, or that the regional prevalence of PASC dysautonomia differs substantially from the distribution of other PASC syndromes.

Findings related to comorbid conditions also present some interesting associations. The finding of comorbid depression or anxiety being associated with a higher risk for PASC has been described before (44). These conditions have also been described as likely comorbid conditions in some dysautonomia syndromes (45). Our analyses found that, consistent with previous research, comorbid depression and anxiety were associated with development of PASC dysautonomia among those diagnosed with COVID-19. Interestingly, we found those with PASC dysautonomia were less likely to have these comorbid conditions as compared to those with non-PASC dysautonomia. These findings may indicate some difference in the pathophysiology in PASC associated dysautonomia from other non-PASC dysautonomia, raising questions around clinical management. The final finding of interest is of the increased likelihood of PASC dysautonomia among those with prescriptions of alpha agonists and beta blockers prior to diagnosis with COVID-19. This finding is interesting given that these medications are routinely used in the management of dysautonomia conditions (46). It is possible that these patients were being managed symptomatically for an undiagnosed dysautonomia prior to receiving an official diagnosis of this condition, or that a diagnosis of dysautonomia was not entered as an ICD-10 code. It is possible that their COVID infection potentiated these underlying symptoms leading to further engagement with the medical system and receipt of diagnosis. However, interpretations of these findings must be taken cautiously given the low numbers of individuals using these medications (two with PASC dysautonomia on alpha-agonists) (39).

Some of the strengths of this analysis include the broad population of U. S. Military Service members included in the data analysis comprising all identified individuals with documented COVID-19 infection in active duty Service members over the included study period. The design of the study further allowed for analysis not only of risks of PASC dysautonomia among individuals with documented COVID-19 infection, but also the identification of dysautonomia in those never diagnosed with COVID-19, allowing for comparisons of PASC dysautonomia to non-PASC dysautonomia. Additionally, the ability to combine multiple data sources to develop the MRAP dataset allowed for a more comprehensive evaluation of medical, demographic, and Service specific variables for evaluation in our analysis.

There were, however, some key limitations in this study to consider as well. As a study including only active duty military personnel, there are concerns over generalizability to the general U. S. population given the age and sex distribution of active duty military Service members along with a generally high health status (47). It is possible that individuals presenting with potentially debilitating syndromes as might appear in dysautonomia may be deemed unfit for service and not fully captured in this analysis if they were unable to continue their service, although the number of separations in the U. S. Military due to Long COVID is currently described as very low (48). These limitations may have resulted in lower numbers of individuals diagnosed with dysautonomia in our analysis than might be expected in a similarly sizes sample of the general U. S. population. The use of the MDR provides a wealth of information on military medical encounters, however it remains administrative claims data which has significant limitations in interpretability and context of medical encounters. The lack of ability to access clinical documentation and elucidate important factors regarding diagnostic pathways, management, and outcomes for dysautonomia within the MHS may limit assessment of the overall impact of these diagnoses, and potential for biases in time to diagnosis after presentation with key symptoms suggestive of dysautonomia. A limitation in the analysis of this dataset also includes the time of observation of individuals in the study. All included individuals were active duty MHS beneficiaries for at least the time period from January 2020 through June 2023; therefore, individuals who left service prior to June 2023 were not included in the analysis, which could potentially bias the results if those who left the service in this timeframe were meaningfully different than those who did not. Also, while medical records were searched for all included individuals back to 2017, included individuals were not required to be active duty Service members back to this date potentially allowing for biases in length of observation prior to the COVID-19 pandemic. Additional analysis limitations include lack of assessment of interaction terms, and of the impact of repeated COVID-19 infections on dysautonomia status, which some studies have found to be associated with increased incidence of PASC syndromes (49). Finally, the low numbers of overall dysautonomia diagnoses are of concern given the small number of events for certain covariates of interest, however *post hoc* model diagnostics support the use of the *a priori* statistical analysis plan and sensitivity analysis using the Firth’s penalized likelihood approach provided similar results.

Next, steps for future research in this space would ideally integrate direct clinical evaluation and management of research participants to better elucidate underlying pathophysiologic mechanisms of PASC dysautonomia and identify potential treatment algorithms for management of these often-complex patients. Further development in this space is currently ongoing in several studies involved in evaluating and treating autonomic dysfunction as a sequelae of COVID-19, including the NIH RECOVER program for PASC research, and Uniformed Services University COVIVA study (50, 51). Further epidemiologic research should seek to further risk stratify individuals for PASC, and further breakdown risks using a syndromic approach as multiple findings in our analysis of PASC dysautonomia were discordant from other findings of risks for PASC overall. Further analysis within this data could attempt to access patient records to assess keywords and use other advanced textual analysis techniques to provide further insight into the clinical course of PASC dysautonomia within the MHS.

In conclusion, PASC dysautonomia is an emerging condition that appears to have some overlap, but also some significant differences in risk factors from non-PASC dysautonomia. Increased severity of COVID-19 infection appears to be a main driver in risk for PASC dysautonomia, with comorbid depression and anxiety also strongly correlated. Early identification and treatment of acute COVID-19 infection to prevent these PASC events is key, and research must focus on developing pathways for identification, typing, and management of PASC syndromes, including PASC dysautonomia to reduce the burden of these conditions on the population.

## Data Availability

The data analyzed in this study is subject to the following licenses/restrictions: The data that support the findings of this study are available from the United States Defense Health Agency. Restrictions apply to the availability of these data, which were used under federal Data User Agreements for the current study and so are not publicly available. Requests to access these datasets should be directed to dha.ncr.pcl.mbx.data-sharing@health.mil.
