# Supplementary figures and images for: Evaluating the risk and risk factors of dysautonomia as a post-acute sequelae of COVID-19: a secondary analysis of a matched case–control dataset
Source: Front Neurol. 2025 Oct 14;16:1653175. doi: 10.3389/fneur.2025.1653175 (PMC12558787; doi:10.3389/fneur.2025.1653175)

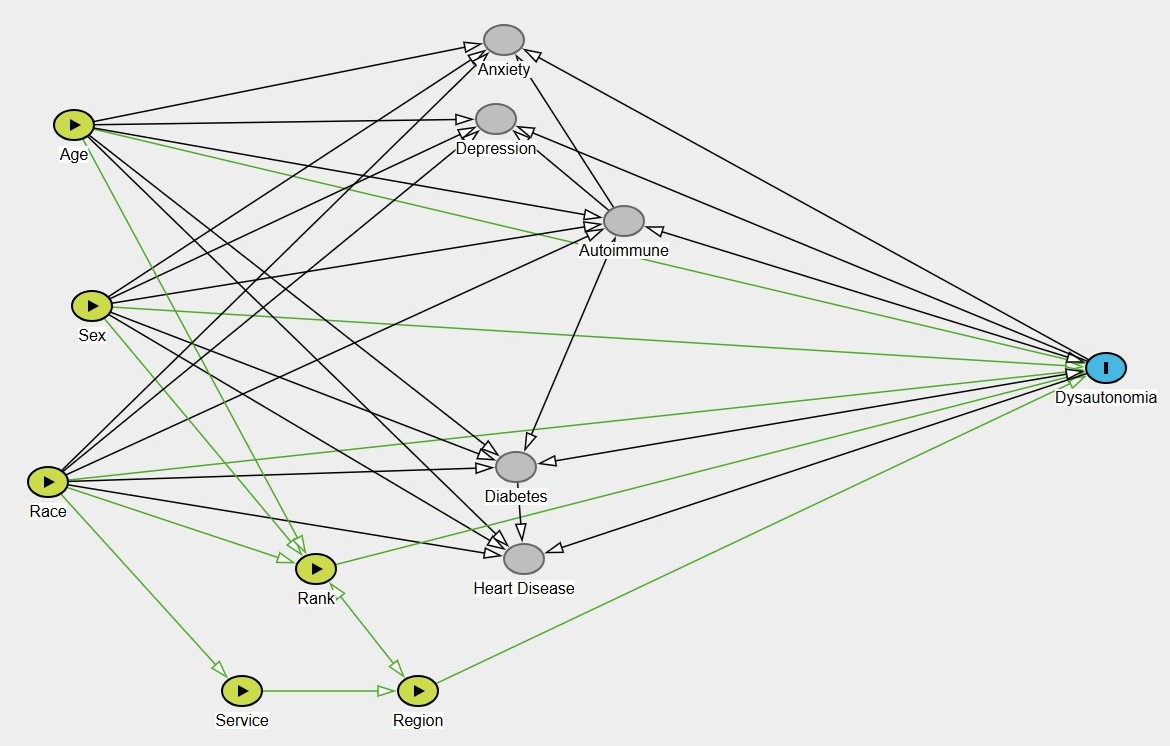

Supplement: Supplementary file 1 [file Image_1.jpeg]

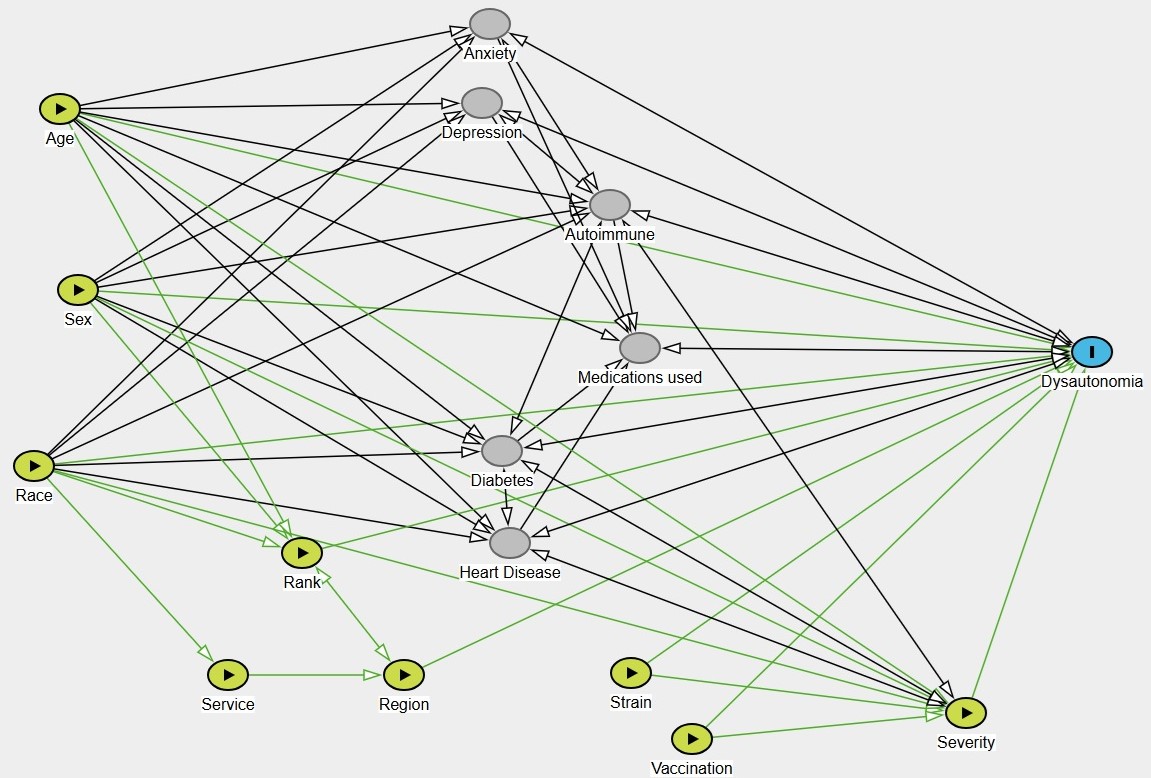

Supplement: Supplementary file 2 [file Image_2.jpeg]
