# Supplementary material for: Evaluating the risk and risk factors of dysautonomia as a post-acute sequelae of COVID-19: a secondary analysis of a matched case–control dataset
Source: Front Neurol. 2025 Oct 14;16:1653175. doi: 10.3389/fneur.2025.1653175 (PMC12558787; doi:10.3389/fneur.2025.1653175)
Supplement: Supplementary file 3 [file Table_1.docx]

| **Code** | **Description** |
| --- | --- |
| U07.1 | 2019‐nCoV acute respiratory disease, COVID‐19, virus identified |
| U07.2 | COVID‐19, virus not identified (clinically diagnosed) |
| J12.82 | Pneumonia due to coronavirus disease 2019 |
| J12.81 | Pneumonia due to SARS‐associated coronavirus |
| B34.2 | Coronavirus, unspecified |
| B97.2 | Coronavirus as the cause of disease classified elsewhere |

Table S1: Codes used to identify individuals with COVID-19 in the M-RAP dataset (in addition to laboratory diagnoses)
