# Supplementary material for: Evaluating the risk and risk factors of dysautonomia as a post-acute sequelae of COVID-19: a secondary analysis of a matched case–control dataset
Source: Front Neurol. 2025 Oct 14;16:1653175. doi: 10.3389/fneur.2025.1653175 (PMC12558787; doi:10.3389/fneur.2025.1653175)
Supplement: Supplementary file 4 [file Table_2.docx]

| **Code** | **Description** |
| --- | --- |
| G90 | Disorders of the autonomic nervous system |
| G90.0 | Idiopathic peripheral autonomic neuropathy |
| G90.4 | Autonomic dysreflexia |
| G90.8 | Other disorders of autonomic nervous system |
| G90.9 | Disorder of the autonomic nervous system, unspecified |
| G90.A | Postural orthostatic tachycardia syndrome [POTS] |

Table S2: ICD-10 codes used to identify dysautonomia in the M-RAP dataset
