# Supplementary material for: Evaluating the risk and risk factors of dysautonomia as a post-acute sequelae of COVID-19: a secondary analysis of a matched case–control dataset
Source: Front Neurol. 2025 Oct 14;16:1653175. doi: 10.3389/fneur.2025.1653175 (PMC12558787; doi:10.3389/fneur.2025.1653175)
Supplement: Supplementary file 5 [file Table_3.docx]

|  | |  | **Full Model** | | |
| --- | --- | --- | --- | --- | --- |
|  |  | | **OR** | **95% CI** | |
| **Sex** | | Male | ref |  |  |
|  |  | Female | 2.442 | 1.738 | 3.415 |
| **Age** | | <25 | 0.960 | 0.581 | 1.586 |
|  |  | 25-34 | ref |  |  |
|  |  | 35-44 | 1.669 | 1.108 | 2.509 |
|  |  | >=45 | 0.828 | 0.317 | 1.841 |
| **Race/**  **Ethnicity** | | American Indian | 0.479 | 0.054 | 1.756 |
|  |  | AAPI* | 0.430 | 0.160 | 0.928 |
|  |  | Black, not Hispanic | 0.326 | 0.182 | 0.547 |
|  |  | Hispanic | 0.577 | 0.358 | 0.891 |
|  |  | White, not Hispanic | ref |  |  |
|  |  | Unknown/Other | 1.005 | 0.456 | 1.934 |
| **Rank** | | Junior Enlisted (E1-E4) | ref |  |  |
|  |  | Senior Enlisted (E5+) | 1.223 | 0.744 | 2.025 |
|  |  | Junior Officer (O1-O3) | 1.363 | 0.700 | 2.561 |
|  |  | Senior Officer (O4+) | 0.879 | 0.370 | 1.965 |
|  |  | Warrant or Other | 0.818 | 0.160 | 2.676 |
| **Service** | | Air Force | ref |  |  |
|  |  | Army | 1.382 | 0.908 | 2.137 |
|  |  | Marine Corps | 2.433 | 1.425 | 4.131 |
|  |  | Navy | 0.977 | 0.586 | 1.622 |
|  |  | Coast Guard / Other | 1.067 | 0.216 | 3.253 |
| **Region**** | | Northeast | ref |  |  |
|  |  | South | 0.677 | 0.352 | 1.198 |
|  |  | Midwest | 0.327 | 0.183 | 0.551 |
|  |  | West | 0.931 | 0.572 | 1.477 |
|  |  | Asia | 0.933 | 0.397 | 1.888 |
|  |  | Europe | 1.112 | 0.525 | 2.098 |
|  |  | Other | 0.862 | 0.236 | 2.217 |
| **Vaccination**  **Status** | | Un/Partial Vaccination |  |  |  |
|  |  | Full Primary Series |  |  |  |
|  |  | At least one booster dose |  |  |  |
| **Variant Era** | | Ancestral | ref |  |  |
|  |  | Delta | 0.544 | 0.315 | 0.893 |
|  |  | Omicron | 0.619 | 0.434 | 0.882 |
| **Severity** | | 1 outpatient visit | ref |  |  |
|  |  | >= 2 outpatient | 1.452 | 0.978 | 2.194 |
|  |  | Hospitalization | 3.141 | 1.292 | 6.691 |
| **Comorbid**  **Conditions** | | Diabetes |  |  |  |
|  |  | Depression | 2.637 | 1.743 | 3.953 |
|  |  | Anxiety | 2.503 | 1.700 | 3.660 |
|  |  | Heart Disease |  |  |  |
|  |  | Autoimmune Disease |  |  |  |
| **Medication Use** | | Alpha Agonist | 10.569 | 2.155 | 31.453 |
|  |  | Beta Blockers | 3.632 | 2.051 | 6.154 |
|  |  | Steroid |  |  |  |
|  |  | SSRI | 1.590 | 1.019 | 2.445 |
|  |  | Stimulant | 4.018 | 1.315 | 9.368 |

Table S3: Sensitivity analysis of full model logistic regression of probable PASC dysautonomia vs. COVID-19 cases without dysautonomia using Firth’s penalized likelihood to assess for rare events effects. *Asian American or Pacific Islander **US regions by HHS (Northeast 1,2,3; South 4,6; Midwest 5,7,8; West 9,10), Other primarily includes Africa and Central/South America
